# Pancreatic Cancer Cells Isolated from Muc1-Null Tumors Favor the Generation of a Mature Less Suppressive MDSC Population

**DOI:** 10.3389/fimmu.2014.00067

**Published:** 2014-02-24

**Authors:** Amritha Kidiyoor, Jorge Schettini, Dahlia Marie Besmer, Stephen Lee Rego, Sritama Nath, Jennifer Marie Curry, Lopamudra Das Roy, Didier Dréau, Pinku Mukherjee

**Affiliations:** ^1^Department of Biology, University of North Carolina at Charlotte, Charlotte, NC, USA

**Keywords:** pancreatic cancer, mucin 1, MDSC, immune suppression, COX-2

## Abstract

Mucin 1 (MUC1) is a transmembrane mucin glycoprotein that is over-expressed and aberrantly glycosylated in >80% of human pancreatic ductal adenocarcinoma (PDA) and is associated with poor prognosis. To understand the role of MUC1 in PDA, we have recently developed two mouse models of spontaneous PDA, one that expresses full-length human MUC1 transgene (KCM mice) and one that is null for MUC1 (KCKO mice). We have previously reported that KCM mice express high levels of myeloid derived suppressor cells (MDSCs) in their tumors and develop highly aggressive PDA. To further understand the underlying mechanism for high MDSC levels in KCM-tumors, we generated primary cell lines from KCM and KCKO-tumors. In this study, we report that MDSCs derived using KCM cells express significantly higher levels of arginase 1 and inducible nitric oxide synthase (markers associated with immune suppression) and lower levels of CD115 (a marker associated with maturation of myeloid cells) as compared to KCKO-derived MDSCs. Functionally, KCM-derived MDSCs secrete significantly higher levels of urea and nitric oxide (NO) when co-cultured with normal splenic cells as compared to KCKO-derived MDSCs. Data indicates that KCM-derived MDSCs remain immature and are more suppressive as compared to KCKO-derived MDSCs. This was further corroborated *in vivo* where MDSCs isolated from KCM-tumor-bearing mice retained their immature state and were highly suppressive as compared to MDSCs derived from KCKO-tumor-bearing mice. Finally, we show that KCM cells secrete significantly higher levels of prostaglandin E2 (PGE_2_), a COX-2 metabolite and a known driver of suppressive MDSCs as compared to KCKO cells. Thus, inhibiting PGE_2_ with a specific COX-2 inhibitor reverses the immunosuppressive and immature phenotype of KCM-derived MDSCs. This is the first report that clearly suggests a functional role of pancreatic tumor-associated MUC1 in the development of functional MDSCs.

## Introduction

Every year, an estimated 270,000 deaths occur worldwide due to pancreatic cancer (1), and in the US, the 1- and 5-year survival rates for all stages combined are 25 and 6%, respectively (2). Therapies that are successful in treating other malignancies are relatively ineffective for pancreatic cancer possibly because cells evade immune recognition (3). Cancer cells release pro-inflammatory factors that promote the generation of immune suppressor cells including myeloid derived suppressor cells (MDSCs) (3). MDSCs play a prominent role in tumor induced immune suppression and are one of the major factors limiting the efficacy of immune therapies (4, 5).

Pancreatic ductal adenocarcinoma (PDA) is the most common type of pancreatic cancer. MUC1 (CD227), a transmembrane mucin glycoprotein is aberrantly over-expressed in >80% of PDA. This suggests a pivotal role for MUC1 in the progression of PDA. MUC1 plays a role in inflammation (6) and cancer progression (7) and has recently been identified as the second most targetable tumor-associated antigen by the National Cancer Institute (8). Indeed, 64% of the tumors diagnosed every year in the United States have aberrant MUC1 expression (7). MUC1 regulates multiple pro-tumor activities including proliferation (9), epithelial to mesenchymal transition (10), and inhibition of apoptosis (11) in tumor cells. However, its role in tumor induced immune suppression and specifically in the generation of MDSCs is not well known.

We were the first to report that in a mouse model of spontaneous PDA (12), overexpression of human MUC1 transgene (PDA.MUC1 mice) significantly enhanced tumor progression and metastasis and showed increased levels of MDSCs and T-regulatory cells (T_regs_) as well as prostaglandin E_2_ (PGE_2_) within the developing tumor (13). In contrast, PDA mice that lacked Muc1 (PDA.Muc1-null mice) had stable disease and the pre-neoplastic lesions did not progress to invasive adenocarcinomas (9, 10). The findings strongly suggest a role for MUC1 in tumor induced immune suppression; possibly via the generation of suppressive MDSCs.

Myeloid derived suppressor cells consist of a heterogeneous population of immature myeloid cell types (14). Generally, these immature myeloid cells differentiate further into mature granulocytes, macrophages, and dendritic cells. However, in cancer, inflammation, trauma, or sepsis, this differentiation process is halted and the myeloid cells remain restricted to their immature state (15). Specifically, tumor derived soluble factors (TDSFs) secreted by tumor cells alter MDSC generation, differentiation, recruitment, and function. TDSFs skew the differentiation of hematopoietic stem cells (HSC) partially hindering maturation of the myeloid lineage (15). In these conditions, MDSCs are found to accumulate in the BM, spleen, blood, lymph nodes, and within tumors (16, 17). TDSFs including PGE_2_ (18, 19), GM-CSF (20), and IL-10 (21) have been implicated in the induction of MDSCs. These factors not only lead to MDSC expansion but also MDSC activation. In addition, MDSCs can be activated by activated T-cells through the action of activated T-cell products; IFNγ, TGF-β, IL-4, and IL-13. In mice, MDSCs are characterized by the co-expression of Gr1 and CD11b molecules (22) and can be classified into subsets: monocytic (CD11b^+^Ly6G^−^Ly6C^hi^) and granulocytic (CD11b^+^Ly6G^+^Ly6C^lo^) (23, 24). MDSCs exert their suppressive activity via a number of different mechanisms; l-arginine depletion (25), nitric oxide (NO) up regulation (26), generation of reactive oxygen species (ROS) (24), and other free radicals including reactive nitric oxide species (RNOS) (27). These mechanisms suppress T-cell proliferation and T-cell effector function, thereby inhibiting anti-tumor immune responses.

In the present study, we compared the effects of TDSFs from PDA cells derived from PDA.Muc1-null mice (KCKO) with PDA cells derived from PDA.MUC1 (KCM) on MDSC phenotype and function *in vitro* and *in vivo*. We report substantive differences in the maturation state and suppressive function of the MDSCs derived in the presence of KCM versus KCKO cells/tumors. We also determined that KCM cells expressed significantly higher levels of COX-2 and secreted higher amounts of PGE_2_ as compared to KCKO cells. Thus, MDSC maturation and function may partly depend upon whether the PDA is Muc1^+^ or Muc1-null.

## Materials and Methods

### Cell lines and mice

The KCKO pancreatic cancer cell line was generated from PDA.Muc1-null tumors and the KCM pancreatic cancer cell line was generated from PDA.MUC1 tumors (9). The murine fibroblast cell lines 3T12 and L929 (ATCC, Manassas, VA, USA), served as negative controls and B16 murine melanoma cell line (a gift from Dr. Tony Hollingsworth, University of Nebraska Medical Center) served as the positive control for Gr1^+^CD11b^+^ MDSCs. B16.Neo (transfected with neomycin empty vector) and B16.MUC1 (expressing full-length human MUC1) murine melanoma cells (a gift from Dr. Tony Hollingsworth, University of Nebraska Medical Center), served as alternative MUC1 expressing and MUC1-null cells. All cell lines were maintained in DMEM (Invitrogen, Grand Island, NY, USA) supplemented with 10% FBS (HyClone, Logan, UT, USA) and 1% 1× penicillin/streptomycin (PS; Cellgro, Manassas, VA, USA). All cultures were kept at 37°C in a 5% CO_2_ humidified atmosphere.

Mice (6- to 8-week-old C57BL/6, Jackson Laboratory, Bar Harbor, Maine, USA) were used for harvesting bone marrows (BM) and spleens and for generation of pancreatic tumors *in vivo*. Mice were handled and maintained in accordance with the University of North Carolina at Charlotte Institutional Animal Care and Use Committee (IACUC)-approved protocol.

### Generation of conditioned media

Tumor cells were cultured with DMEM supplemented with reduced serum (2% FBS). Forty-eight hours post-incubation, the tumor cell conditioned media (TCCM) containing the TDSFs released by tumor cells were collected. The TCCMs were centrifuged (3000 rpm, 10 min), filtered (0.2 μm filter, Fisher Scientific, Pittsburgh, PA, USA), and stored at −80°C until use. Fibroblast conditioned media (FCMs) were obtained from 3T12 and L929 fibroblast cells in a similar fashion.

### Celecoxib treatment

KCM cells at 70% confluency were treated with the COX-2 inhibitor Celecoxib (50 μM; Pfizer, New York City, NY, USA) in serum-free DMEM for 48 h. Cells were washed twice with 1× PBS and incubated for an additional 48 h with fresh DMEM supplemented with 2% FBS (reduced serum). TCCMs were collected, and stored as described above.

### Pancreatic tumor growth *in vivo*

1 × 10^6^ cells of 3T12, B16.Neo and B16.MUC1, KCKO, and KCM cells were re-suspended in 100 μL of 1× PBS:Matrigel^®^ (BD Biosciences, San Jose, CA, USA) 1:1 and injected subcutaneously into the flank of C57BL/6 mice. Seventeen days post-injection, the mice were sacrificed and the tumor and organs harvested. The tumors were weighed and tumor lysates were prepared. BM and splenocytes were harvested and used as described below.

### Generation of bone marrow progenitors

Bone marrow cells were obtained from the femurs and tibias of 6- to 8-week-old C57BL/6 mice. BM cells (1 × 10^6^) were cultured in DMEM supplemented with 10% FBS, 1% PS, 10 ng/ml GM-CSF (Peprotech, Rocky Hill, NJ, USA), 10 ng/ml IL-4 (Peprotech, Rocky Hill, NJ, USA), and 50 μM of 2-ME alone or in the presence of 30% v/v TCCM or control (FCM) in 24-well flat bottom plates as described in Ref. (24, 28, 29). Cells were maintained at 37°C in 5% CO_2_ humidified atmosphere for 5 days. On day 3 of culture, floating cells were removed and media replenished with GM-CSF, IL-4, and TCCM/FCM. As BM cultures are considered suppressive by day 5 (28), cells were collected on day 5 and analyzed by flow cytometry. The optimal TCCM concentration (30% v/v) was determined from titration curves of Gr1^+^CD11b^+^MDSCs, this concentration was not associated with any noticeable BM cell death. To account for differing proliferation rates between cells and treatment conditions, BCA analyses were conducted on both cell lysates and conditioned media. The total protein concentrations were similar in the lysates and conditioned media obtained from KCKO, KCM, and KCM cells treated with Celecoxib (data not shown).

### Flow cytometry

Floating bone marrow derived cells (BMDC) and loosely adherent cells from BM cultures were collected, centrifuged, and cell pellet re-suspended in staining buffer (SB; 1× PBS with 1% FBS and 0.1% sodium azide). Cells were stained with anti-Gr1-APC, anti-CD11b-PE, anti-Ly6C-FITC, anti-Ly6G-APC, anti-CD11c-FITC, and biotinylated anti-CD115 for total MDSC population, subsets, and maturation phenotypes, respectively. For the detection of intracellular antigens, the cells were fixed and simultaneously permeabilized with BD Cytofix/Cytoperm and stained with anti-iNOS-FITC and anti-Arg-1 primary antibody and appropriate secondary APC conjugated antibody. Splenocytes were harvested from spleens of 6- to 8-week-old C57BL/6 mice under sterile conditions, red blood cells were lysed (1× RBC lysing buffer) and single-cell suspensions were prepared for staining with anti-Gr1-APC and anti-CD11b-PE antibodies. KCM and KCKO cells were made into single-cell suspensions and stained with anti-MUC1-PE antibody. All antibodies were purchased from BD Biosciences (BD Biosciences, San Jose, CA, USA). Samples were run on a FACSCalibur flow cytometer (BD Biosciences, San Jose, CA, USA) and data analyzed using the FlowJo software (Tree Star Inc., Ashland, OR, USA). Data are expressed as percentage positive cells and/or mean fluorescence intensity (MFI).

### BMDCs collected for nitric oxide and arginase activity assays

Bone marrow derived cells were generated as described above with the following modifications. On day 3, 100 U/ml IFN-γ (Peprotech, Rocky Hill, NJ, USA) and 0.1–1 μg/ml LPS (Sigma, St Louis, MO, USA) were added to enhance the suppressive activity of MDSCs (30). As MDSCs populations can be sorted by presence of Gr1 marker alone and are known to possess suppressive activity on day 5 (31), BMDCs Gr1^+^cells were sorted using anti-APC microbeads (Miltenyi, Bergisch Gladbach, Germany) and anti-Gr1-APC antibody (BD Biosciences, San Jose, CA, USA). The purity of the sorted cell fraction was >90%.

### Splenocyte co-culture for nitric oxide and arginase activity assays

Spleens were harvested from 6- to 8-week-old C57BL/6 mice under sterile conditions. Red blood cells were lysed (1× RBC lysing buffer) and single-cell suspensions were prepared. Gr1^+^ cells isolated from BM cultures were added to 2 × 10^5^ naïve C57BL/6 splenocytes per well in 96-well flat bottom plates at 1:1, 1:2, and 1:4 ratios. These co-cultures were stimulated with 1 μg/ml anti-CD3 antibody (BD Biosciences, San Jose, CA, USA). Following a 72-h incubation, supernatants and cell lysates were collected. The supernatants were assessed for NO production and the cell lysates were used to determine arginase activity. Gr1^+^ cells from BM cultures of tumor-bearing mice were incubated with freshly isolated splenocytes at the optimal ratio of 1:1 for the NO and urea activity assays. Anti-CD3 antibody was added to stimulate splenocytes as MDSCs are activated and produce NO and urea only in the presence of activated/stimulated T-cells.

### Nitric oxide production

Equal volumes of culture supernatants were mixed with Griess reagent (1% sulfanilamide in 5% phosphoric acid and 0.1% *N*-1-naphthylethylenediamine dihydrochloride in double-distilled water; Promega, Fitchburg, WI, USA) and incubated at room temperature for 10 min. The absorbance was measured at 550 nm using a BioTek microplate reader (BioTek, Winooski, VT, USA). Nitrate concentrations (μM) were determined by comparing sample OD values to a standard curve generated by serial dilution of 0.1 mM sodium nitrite.

### Arginase activity

Gr1^+^ cells were lysed for 30 min with 100 μl of 0.1% Triton X-100. Subsequently, 100 μl of 25 mM Tris–HCl and 10 μl of 10 mM MnCl_2_ were added. The arginase enzyme was activated by heating the lysate for 10 min at 56°C. 100 μl of 0.5 M l-arginine (pH 9.7) substrate was added to the lysate and incubated for 120 min at 37°C. Reaction was stopped with 900 μl of acid mixture 1 H_2_SO_4_ (96%):3 H_3_PO_4_ (85%):7 H_2_O. 40 μl of α-isonitrosopropiophenone (dissolved in 100% ethanol) was added to the mixture and heated for 30 min at 95°C. Absorbance was measured at 540 nm. The urea concentrations (μM) were determined by comparing sample OD values to urea standards generated by serial dilution of 1 mM urea solution.

### Lymphocyte proliferation assay

To monitor lymphocyte proliferation in the presence of Gr1^+^ MDSCs, single-cell suspensions of splenocytes harvested from 6- to 8-week-old C57BL/6 mice were stained using the CellTrace CFSE Cell Proliferation Kit (Molecular Probes, Eugene, OR, USA). The splenocytes were centrifuged at 2000 rpm for 2 min to obtain a pellet. The pellet was re-suspended in pre-warmed PBS with 0.1% BSA containing the probe at a final working concentration of 10 μM to a final concentration of 1 × 10^6^ cells/ml. Cells were incubated at 37°C for 15 min, and then pelleted by centrifugation at 2000 rpm for 2 min. Labeled cells were re-suspended in pre-warmed media, and incubated for 30 min at 37°C. The cells were then pelleted again by centrifugation and washed twice before being re-suspended in media supplemented with serum at the desired concentration. To measure the fluorescence at Day 0, 0.5 × 10^6^ cells were kept aside. After 3 days the fluorescence was determined. CFSE dilution was analyzed using FACSCalibur and further analyzed with the FlowJo software. Data was expressed in terms of a proliferation index wherein MFI values of CSFE fluorescence are normalized to Day 0 CFSE fluorescence of splenocytes.

### Western blots and ELISA

Protein lysates (30 μg per sample) were separated by SDS-PAGE and transferred to PVDF membrane for immunoblotting. Anti-COX-2, anti-MUC1 TR (extracellular portion of MUC1), anti-MUC1 CT (cytoplasmic tail of MUC1), and anti-IL-10 antibodies (SantaCruz Biotechnology, Dallas, TX, USA) were used.

PGE_2_ ELISAs were performed using the Prostaglandin E_2_ EIA Kit-Monoclonal kit (Cayman Chemical; Ann Arbor, MI, USA) on TCCM and FCM. PGEM (13,14-dihydro 15-keto PGA_2_) ELISAs were performed using the Prostaglandin E Metabolite EIA Kit (Cayman Chemical; Ann Arbor, MI, USA) on tumor lysates and serum from tumor-bearing mice. PGE_2_ is metabolized rapidly *in vivo* and hence an accurate measurement of PGE_2_ is not possible from *in vivo* samples, therefore the PGE_2_ metabolite (PGEM) concentration is assessed in the serum and tumor lysate of tumor-bearing mice.

### Proteomics

KCM and KCKO-tumor lysates from tumor-bearing mice were electrophoretically separated, gel slices digested with trypsin and analyzed using a Nano Acuity UPLC system connected to an LTQ-Orbitrap hybrid MS system with a Nanospray interface. Data were collected using Xcaliber and processed using Bioworks software and mouse v.3.18 FASTA database. The following SEQUEST parameters were used: mass tolerance of 0.01 Da for precursor ions and 0.5 Da for fragment ions, variable modification on methionine of 16 Da, and maximum missed cleavage of 1. Search results were entered into Scaffold software (Proteome Software; Portland, OR, USA) for compilation, normalization, and comparison of spectral counts. Protein identifications were made at the peptide probability of 95% and protein probability of 99%. Experiments for Mass spectroscopy were duplicated for a power law global error model (PLGEM). Datasets were imported into the R program for statistical computing. Data are displayed as fold increase in KCM cells/tumors as compared to KCKO cells/tumors.

### Statistical analyses

All data are expressed as mean ± SEM. Differences between conditions tested were determined by one-way ANOVA followed by Tukey’s *post hoc* test using GraphPad Prism 5.0 software (La Jolla, CA, USA). The functional assays were analyzed by two-way ANOVA followed by Bonferroni post-test using GraphPad Prism 5.0 software. Probability values of *p* ≤ 0.05 were considered significant.

## Results

### TDSFs from MUC1 expressing PDA cells favor the expansion of monocytic MDSCs

**Figure 1:**
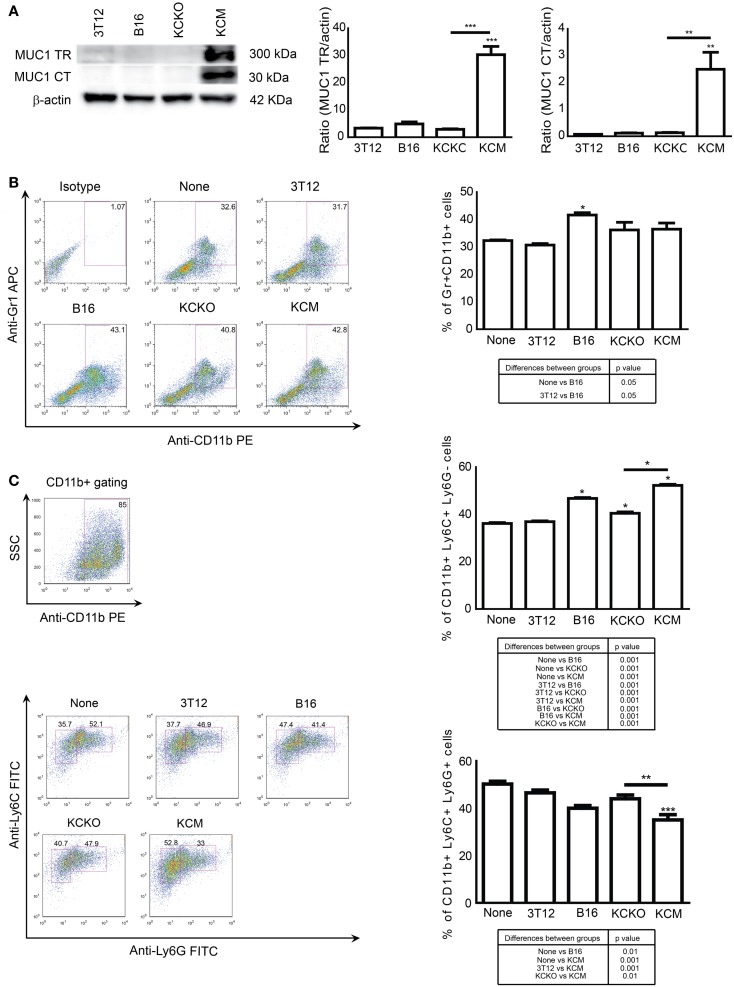
**Generation of MDSCs in the presence of various TDSFs *in vitro***. BM cells were harvested from 6- to 8-week C57BL/6 mice (*n* = 4), cultured for 5 days with GM-CSF, IL-4, and with or without 30% v/v TCCM/FCM. Cells were stained with anti-Gr1, anti-CD11b, anti-Ly6C, and anti-Ly6G antibodies and analyzed by flow cytometry. **(A)** Western blot analysis and quantitative densitometry of the protein expressions of MUC1 probed with anti-MUC1 TR (detects extracellular motif of MUC1) and anti-MUC1 CT (detects cytoplasmic tail motif of MUC1) antibodies on cell lysates. MUC1 protein expression normalized to β-actin protein expression. Western blotting for MUC1 and β-actin protein expression was carried out three times; **(B)** Representative dot plot and graph of percent Gr1^+^CD11b^+^ cells; **(C)** Representative dot plot and graph of percent CD11b^+^Ly6C^+^LyG^−^ and CD11b^+^Ly6C^+^Ly6G^+^ cells. The MDSC subsets were obtained by gating on CD11b^+^ cells as indicated (top left plot). Percentages are not absolute numbers. Mean and standard error are plotted. *p < 0.5, **p < 0.01, and ***p < 0.001 for statistically significant differences from control (3T12) levels unless indicated by the line above the bars. A table of statistically significant differences between each group is provided below dot plots.

Bone marrow cells were cultured in media supplemented with GM-CSF and IL-4 with or without 30% v/v TCCM from KCM or KCKO to determine whether MUC1 expression in PDA cells affects MDSC expansion from BM cells. MUC1 expression was determined by western blotting using two antibodies, one to the MUC1 extracellular tandem repeat domain (MUC1 TR, >250 kDa) and one to the cytoplasmic tail domain (MUC1 CT, <30 kDA). KCM cells express high levels of MUC1 while KCKO, B16 (positive control), and 3T12 (negative control) cells do not (Figure 1A). On day 5 of BM culture, Gr1^+^CD11b^+^ cells were analyzed by flow cytometry. With no TCCM (none) and FCM from 3T12 cells, ~30% of the BM cells were Gr1^+^CD11b^+^ (Figure 1B) confirming previous observations (24). BM cells cultured with B16 TCCM generated >40% Gr1^+^CD11b^+^ cells also corroborating previous reports (24). BM cells cultured with KCM TCCM and KCKO TCCM also generated ~40% Gr1^+^CD11b^+^ cells. However, no difference was noted in the Gr1^+^CD11b^+^ population between KCM and KCKO TCCM (Figure 1B).

Next, we examined whether the presence of MUC1 in PDA cells influences the differential expansion of the MDSC subsets, i.e., monocytic versus granulocytic (24, 32). Cells were gated on CD11b^+^ population and the two subset populations were analyzed based on presence/absence of Ly6G. A significant increase in the monocytic subset (CD11b^+^Ly6G^−^Ly6C^+^, ~50 versus 35%, *p* < 0.05) and a corresponding decrease in the granulocytic subset (CD11b^+^Ly6G^+^Ly6C^+^, ~35 vs. ~45%, *p* < 0.01) was observed in the BM cells treated with TCCM from KCM cells compared to TCCM from KCKO cells (Figure 1C). Significantly, fewer monocytic MDSCs were induced when BM cells were cultured with 3T12 FCM compared to TCCM from B16 or KCM cells (Figure 1C).

### Expression of MUC1 by PDA cells hinders MDSC maturation

**Figure 2:**
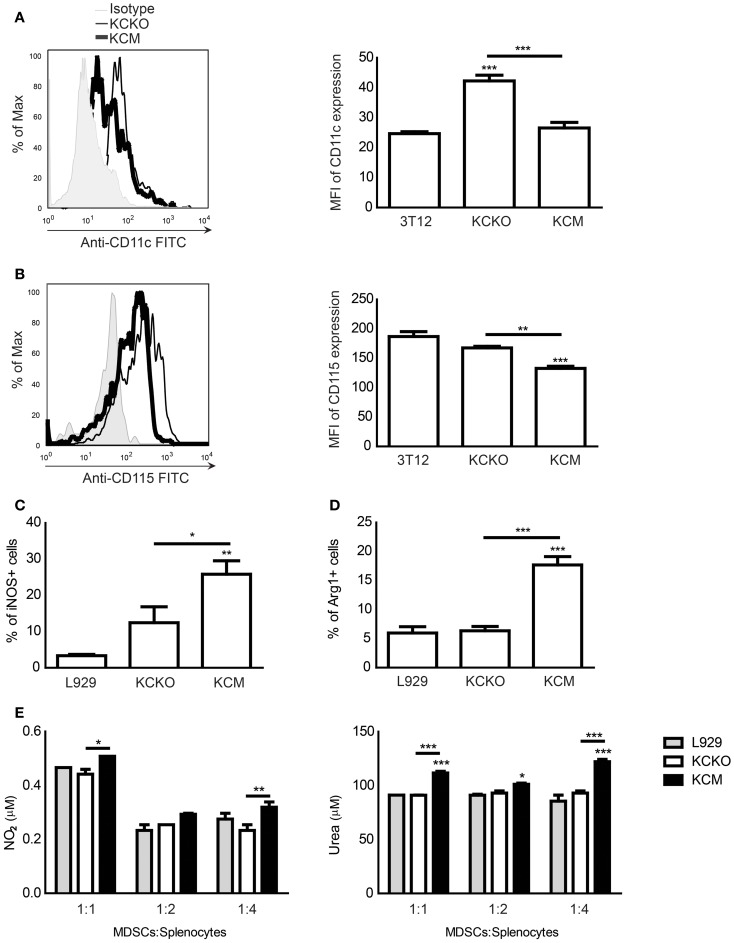
**Analysis of maturation and suppression markers on MDSCs in response to various TDSFs *in vitro***. BM cultures were established as described (*n* = 4 mice). Cells were stained with anti-Gr1, anti-CD11b, anti-CD11c, anti-CD115, anti-iNOS, and anti-Arg-1 antibodies and analyzed by flow cytometry. **(A)** CD11c expression on CD11b^+^ gated cells treated with various TCCM; **(B)** CD115 expression on CD11b^+^ gated cells treated with various TCCM. Results are expressed as mean fluorescence intensity (MFI) along with representative histogram. Mean and standard error are plotted. ***p < 0.001 for statistically significant differences from control (3T12) levels and **p < 0.01 for CD115 MFI differences between KCKO TCCM and KCM TCCM; **(C)** Percent of iNOS^+^ cells on Gr1^+^CD11b^+^ gated cells treated with various TCCM; **(D)** Percent of Arg-1^+^ cells on Gr1^+^CD11b^+^ gated cells treated with various TCCM. Percentages are not absolute numbers; **(E)** Gr1^+^ cells from BM cultures were co-cultured with freshly isolated splenocytes stimulated with anti-CD3 for 3 days at ratios of 1:1, 1:2, and 1:4 for 72 h. The nitric oxide concentration was assessed in the co-culture supernatant and the urea concentration was assessed in the cell lysate. One representative experiment out of three is shown. Mean and standard error are plotted. *p < 0.05, **p < 0.01, and ***p < 0.001 for statistically significant differences from control (L929) levels unless indicated by the line above the bars.

It is known that less mature MDSCs are more suppressive than mature MDSCs (24, 33). Thus, we assessed the maturation state of the MDSCs. An array of maturation and activation markers including CD11c, CD115, CD80, CD86, F4/80, CD40, MHC class I, MHC class II, B7H-1, and B7H-4 were analyzed. There was no change in CD80, CD86, F4/80, CD40, MHC class I or II, B7H-1, and B7H-4 levels (data not shown). However, we did observe a significant upregulation in CD11c expression on the CD11b^+^ population when BM cells were cultured in the presence of TCCM from KCKO cells (Figure 2A) but not from KCM cells. CD11c (integrin alpha X chain) is one of the markers of MDSC maturation and is used to describe a subpopulation of mature MDSCs (34). Second, we noted a significant down-regulation of CD115 expression on the CD11b^+^ population when BM cells were cultured with TCCM from KCM cells but not KCKO cells (Figure 2B). CD115 is a receptor for colony-stimulating factor 1 (CSF-1) and is expressed on monocytes, macrophages, and macrophage/dendritic cell precursors and is a marker of mature myeloid cells (35). It is important to note that of all the maturation and activation markers assessed, a notable change was only observed in two markers. These markers are myeloid maturation markers while the others are myeloid activation markers indicating that TCCM from KCKO cells may promote maturation of MDSCs while TCCM from KCM cells hinders its maturation.

### Expression of MUC1 in PDA cells promotes a suppressive MDSC phenotype

We investigated whether the KCM-derived MDSCs were more suppressive than the KCKO-derived MDSCs by evaluating the expressions of iNOS and Arg-1 along with the concentrations of their downstream byproducts, NO and urea, respectively. The intracellular enzymes iNOS and Arg-1 are strongly associated with MDSC-mediated suppression (4) and elevated levels of NO and urea are produced by activated suppressive MDSCs (24). Significant increases in the expression of iNOS^+^ and Arg-1^+^ cells were observed within the KCM-derived versus KCKO-derived Gr1^+^CD11b^+^ MDSC population (Figures 2C,D). To assess the concentration of NO and urea, Gr1^+^ cells (MDSCs) were sorted from BM cultures treated with TCCM or FCM and co-cultured with varying ratios of activated splenocytes for 3 days. Significantly, higher concentrations of NO and urea were produced by KCM-derived MDSCs as compared to KCKO-derived MDSCs (Figure 2E).

### KCM-tumor-bearing mice exhibit higher levels of MDSCs that retain their immature state

**Figure 3:**
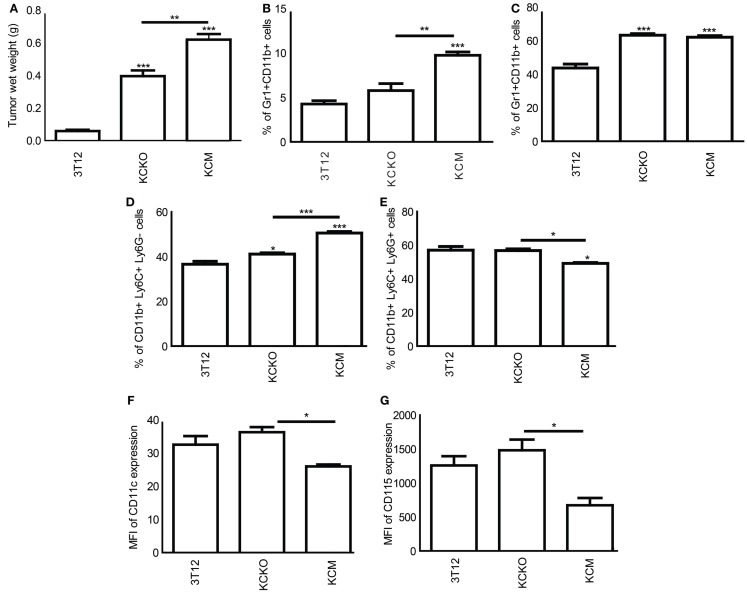
**Characterization of MDSCs in tumor-bearing mice: C57BL/6 mice (*n* = 4) were injected in the flank with 1 × 10^6^ 3T12, KCM, and KCKO cells**. Upon sacrifice, the tumor, spleen, and bone marrow were harvested. BM cultures were established as previously described. BMDCs were stained with anti-Gr1, anti-CD11b, anti-Ly6C, anti-Ly6G, anti-CD11c, anti-CD115 antibodies, and analyzed as described previously. **(A)** Tumor wet weight in grams; **(B)** Percentage of Gr1^+^CD11b^+^ cells in the spleen; **(C)** Percentage of Gr1^+^CD11b^+^ cells in the bone marrow **(D,E)** Percentage of MDSC subsets (CD11b^+^Ly6C^+^LyG^−^ and CD11b^+^Ly6C^+^Ly6G^+^) in the bone marrow; **(F,G)** CD11c and CD115 expression analyzed on CD11b^+^ gated cells from bone marrow expressed in terms of MFI. Percentages are not absolute numbers. Mean and standard error are plotted. *p < 0.05, **p < 0.01, and ***p < 0.001 for statistically significant differences from control (3T12) levels unless indicated by line above the bars.

To recapitulate the *in vitro* data in an *in vivo* setting, C57BL/6 mice were injected subcutaneously with 3T12, KCKO, and KCM cells. At time of euthanasia (17 days post tumor challenge), tumors were dissected and weighed. KCM-tumors were significantly larger than the KCKO or 3T12 tumors (Figure 3A), matching previous observations reported by our group (9).

Significantly, higher levels of Gr1^+^CD11b^+^ MDSCs were observed in the spleen of KCM-tumor-bearing mice as compared to KCKO or 3T12-tumor-bearing mice (Figure 3B). Although no change in percent of MDSC was observed in the BM of KCM versus KCKO-tumor-bearing mice (Figure 3C), the numbers of monocytic and granulocytic populations were significantly higher and lower, respectively, in the KCM compared to KCKO and 3T12-tumor-bearing mice (Figures 3D,E) corroborating the *in vitro* data (Figure 1C). Moreover, BM-derived MDSCs from KCM-tumor-bearing mice had lower expression of CD11c and CD115 as compared to MDSCs from KCKO-tumor-bearing mice (Figures 3F,G). Thus, data clearly suggests that MDSCs from KCM-tumor-bearing mice retain their immature state *in vivo*.

### MDSCs from KCM-tumor-bearing mice are immunosuppressive

**Figure 4:**
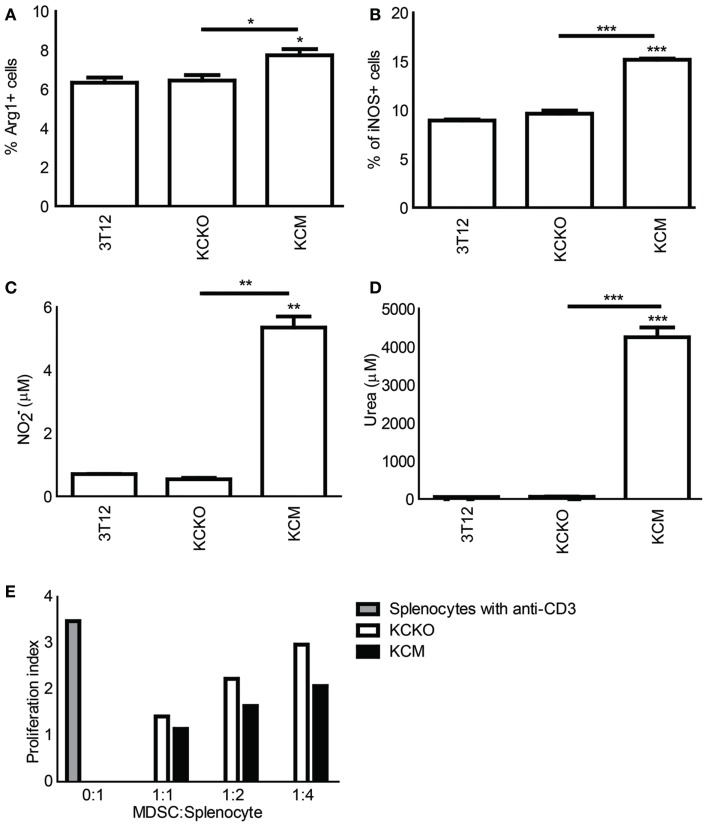
**Analysis of suppressive markers on MDSCs in tumor-bearing mice: C57BL/6 mice (*n* = 4) were injected in the flank with 1 × 10^6^ 3T12, KCM, and KCKO cells**. BM cultures were established as previously described. BMDCs were stained with anti-CD11b, anti-iNOS, and anti-Arg-1 antibodies and analyzed as described previously. **(A,B)** Percentages iNOS^+^ and Arg-1^+^ cells on Gr1^+^CD11b^+^ BM gated cells. Percentages are not absolute numbers; Splenocytes were isolated from naïve C57BL/6 mice and co-cultured at 1:1 ratio with Gr1^+^ cells isolated from BM cultures from tumor-bearing mice for NO and urea production analyses. **(C,D)** Concentrations of NO and urea were assessed in the supernant and cell lysate respectively of freshly isolated splenocytes stimulated for 3 days with anti-CD3 in the presence or absence of sorted Gr1^+^ cells from BM cultures of tumor-bearing mice. Mean and standard error are plotted. For **(A,B)** *p < 0.05, **p < 0.01, and ***p < 0.001 for statistically significant differences from control (3T12) levels unless indicated by line above the bars; **(E)** CFSE labeled splenic cells were stimulated for 3 days with anti-CD3 in the presence or absence of sorted Gr1^+^ cells at varying ratios from BM cultures of tumor-bearing mice to assess lymphocyte proliferation. Data is expressed in terms of proliferation index (all CSFE MFI were normalized to Day 0 CFSE fluorescence of splenocytes). Data is representative of one of three experiments. Due to technical issues, there were no biological replicates for this experiment and hence a two-way ANOVA could not be performed. However, a paired t-test was conducted between KCKO and KCM MFI values for equivalent dilutions. The difference was found to be significant (*p* ≤ 0.05) at each dilution (not shown).

We assessed the expression of iNOS and Arg-1 on the MDSCs from the BM of tumor-bearing mice. The percent of Gr1^+^CD11b^+^ MDSCs expressing iNOS and Arg-1 were significantly higher in KCM as compared to KCKO and 3T12-tumor-bearing mice (Figures 4A,B). Further, when BM-derived MDSCs from KCM-tumor-bearing mice were co-cultured with varying ratios of activated splenocytes, higher concentrations of NO and urea were produced (Figures 4C,D) at optimal co-culture ratios of 1:2 for NO and 1:1 for urea. MDSCs from KCKO mice failed to produce NO or urea above basal levels (Figures 4C,D). In addition, data shown in Figure 4E suggests that BM-derived MDSCs from KCM-tumor-bearing mice may be capable of suppressing lymphocyte proliferation in a dose dependant manner to a greater extent than BM-derived MDSCs from KCKO-tumor-bearing mice. For this experiment, MDSCs had to be pooled from *n* = 3 mice due to limited numbers of MDSCs obtained after sorting to conduct the co-culture. Therefore, technical replicates were done and *p*-values could not be generated using the two-way ANOVA statistical test. However, a paired *t*-test between KCKO and KCM MFI values was done for equivalent dilutions. The difference was found to be significant (*p* ≤ 0.05) at each dilution. A representative of three experiments is shown in Figure 4E.

### KCM cells express significantly higher levels of IL-10, COX-1, and 2, PGE synthase 2 and 3, and release higher levels of PGE_2_ than KCKO cells

**Figure 5:**
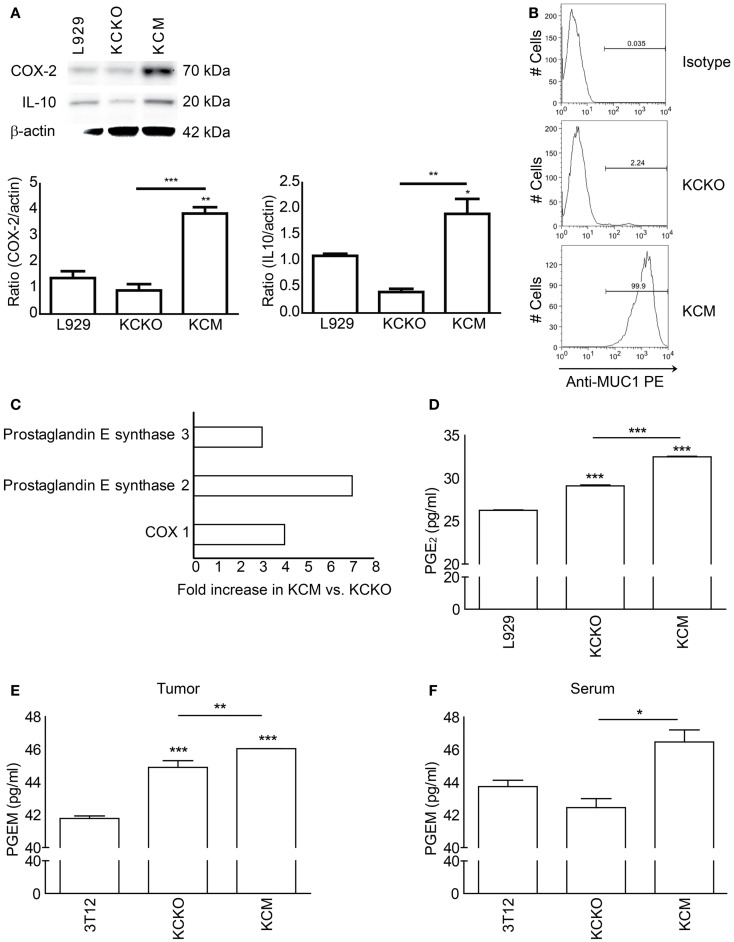
**Analysis of TDSFs from KCM and KCKO cells involved in MDSC-mediated suppression**. Whole cell lysate, conditioned media, tumor lysate, and serum were analyzed for common immune modulating factors. **(A)** Whole cell lysates (30 μg) of KCM and KCKO cells were loaded onto SDS gels and blotted with anti-COX-2, anti-IL-10, and anti-β-actin. The quantitative densitometry of the protein expressions of COX-2 and IL-10 normalized to β-actin is shown as well. Western blotting for COX-2, IL-10, and β-actin protein expression was carried out three times; **(B)** MUC1 expression in KCKO and KCM cells determined by flow cytometry. The gate for percentage positive MUC1 cells was set using the appropriate isotype control; **(C)** Proteins over-expressed in KCM versus KCKO-tumor cell lysates from tumor-bearing mice determined by proteomics analyses (Data expressed in fold changes); **(D)** PGE_2_ concentrations in conditioned media of KCM and KCKO cells measured by ELISA; **(E,F)** PGEM (13,14-dihydro-15-keto PGA_2_) concentrations quantified by ELISA in serum and tumor lysate from tumor-bearing mice (*n* = 4). Mean and standard error plotted. *p < 0.05, **p < 0.01, and ***p < 0.001 for statistically significant differences from control levels unless indicated by the line above the bars.

To determine the mechanism(s) underlying the MUC1 driven generation of suppressive MDSCs, we assessed various immunosuppressive factors (TDSFs) implicated in MDSC generation. We detected significantly higher levels of COX-2 and IL-10 by western blotting in KCM as compared to KCKO and L929 cell lysates (Figure 5A). In Figure 5B, we confirm the presence and absence of MUC1 by flow cytometry in KCM and KCKO from which lysates were made and mice were injected with. In addition, the proteomics data revealed significant fold increases in COX-1 (fourfold), PGE synthase 2 (sevenfold), and PGE synthase-3 (threefold) in KCM versus KCKO-tumors (Figure 5C). These enzymes are involved in prostaglandin metabolism and are known to influence MDSC generation, recruitment, and function in cancer.

Further, the concentration of PGE_2_, the major byproduct of the COX enzyme activities was significantly higher in the KCM TCCM compared to the KCKO and 3T12 TCCM (Figure 5D). Similarly, levels of PGE metabolite (PGEM; 13,14-dihydro-15-keto PGA_2_) were significantly higher in the tumors and serum of mice bearing the KCM-tumor compared to KCKO and 3T12 tumors (Figures 5E,F).

### Inhibition of COX-2 reverses MUC1 driven effects on MDSC maturation and function

**Figure 6:**
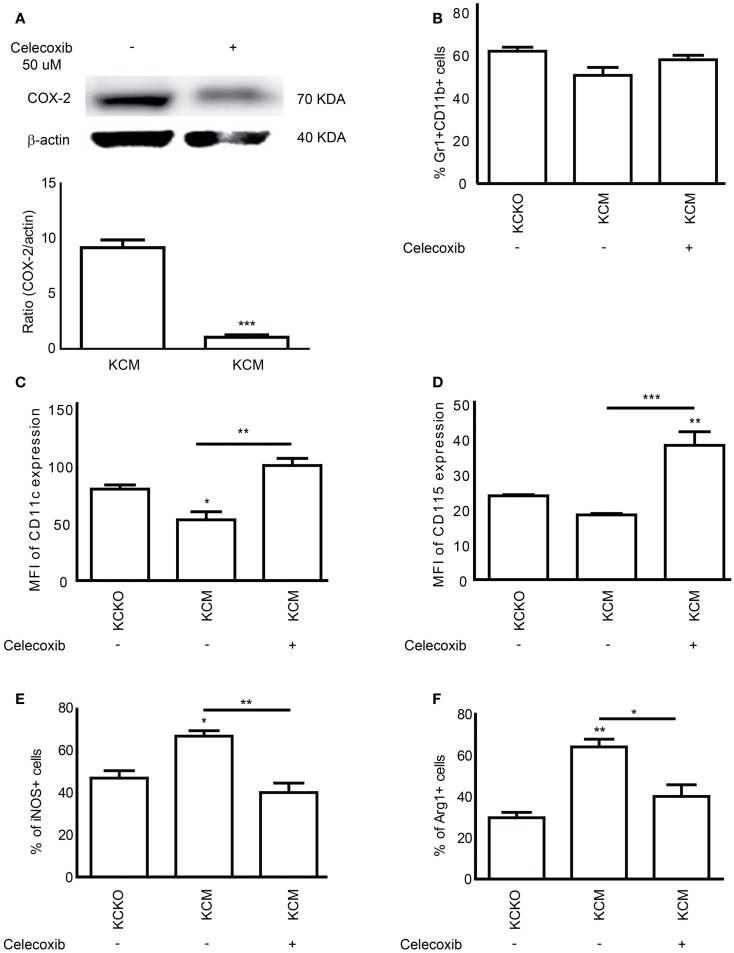
**COX-2 inhibitor Celecoxib reverses the suppressive phenotype of MDSCs**. BM cells (*n* = 4) were cultured with 30% v/v TCCM from KCKO, KCM, and celecoxib-treated KCM cells (with 50 μM Celecoxib). Cells were stained with anti-Gr1, anti-CD11b, anti-CD11c, anti-CD115, anti-iNOS, and anti-Arg-1 antibodies. **(A)** Western blot analysis and quantitative densitometry of the protein expression of COX-2 gene on whole cell lysates from KCM and celecoxib-treated KCM cells. COX-2 protein expression was normalized for β-actin protein expression. Western blotting for COX-2 and β-actin protein expression was carried out 3 times; **(B)** Percentage of Gr1^+^CD11b^+^ MDSC population; **(C,D)** Expression of CD11c and CD115 expression on Gr1^+^CD11b^+^ cells; **(E,F)** Percentage of iNOS^+^ and Arg-1^+^ cells on Gr1^+^CD11b^+^ gated cells. Percentages are not absolute numbers. Mean and standard error are shown. *p < 0.05, **p < 0.01, and ***p < 0.001 for statistically significant differences from KCKO levels unless indicated by the line above the bars.

To determine if indeed MUC1 driven effects on MDSC maturation and suppression were mediated by COX/PGE_2_, we treated KCM cells with the selective inhibitor of COX-2 (Celecoxib). KCM cells were treated with 50 μM of Celecoxib for 48 h and TCCM collected. Treatment with Celecoxib significantly reduced the expression of COX-2 in KCM cells as determined by western blot analyses (Figure 6A). When BM cells were cultured with TCCM from KCKO, KCM, and Celecoxib-treated KCM cells, we observed no differences in the numbers of total Gr1^+^CD11b^+^ cells (Figure 6B). However, a significant increase was observed in the expression of the maturation markers (CD11c and CD115) in MDSCs derived using TCCM from Celecoxib-treated KCM cells (Figures 6C,D). In addition, a significant decrease in the percentages of iNOS^+^ and Arg-1^+^ cells was observed in MDSCs derived with TCCM from Celecoxib-treated KCM cells, approaching the levels observed with KCKO TCCM (Figures 6E,F). These findings strongly indicate that inhibiting the COX-2 pathway enables the immature KCM-induced MDSCs to undergo maturation and in turn lose their suppressive phenotype.

## Discussion

Patients with pancreatic cancer display high levels of circulating tumor MUC1 (CA19-9) that correlate with poor prognosis and disease recurrence (36). In addition, these patients exhibit significant immune dysfunction characterized by increased levels of immune suppressor cells including T_regs_ and MDSCs (37, 38). Although, tumor-associated MUC1 has been reported to be immunosuppressive (39, 40), its role in MDSC generation and function in pancreatic cancer has never been explored. We recently reported that tumors from PDA mice that over express human MUC1 have elevated levels of MDSCs and favor a highly immune suppressive and pro-inflammatory microenvironment (13).

In this study, we report that there was a significant increase in total MDSC numbers in the spleen of KCM versus KCKO-tumor-bearing mice but no difference in the BM (Figure 3). This implies that KCM-tumor may have a profound effect on the recruitment of MDSCs to the lymphoid organs but not on its differentiation in the BM. Further, we show *in vitro* that TCCM from KCM cells promote the expansion of a monocytic MDSC subset, that are immature and more suppressive than MDSCs derived using TCCM from KCKO cells (Figure 1). Interestingly, when the individual MDSC subsets were further analyzed for presence of maturation and suppressive markers, lower levels of CD11c and CD115 was observed in both the monocytic and granulocytic MDSC subsets (Figures S1C–F in Supplementary Material). However, an increase in iNOS^+^ cells was predominantly in the monocytic subset while an increase in Arg-1^+^ cells was predominantly in the granulocytic subset (Figures S1G–J in Supplementary Material). The observations that the suppressive activity of MDSCs is primarily conferred by the monocytic subset and that as MDSCs mature, they lose their suppressive capability is supported by several other published studies (24, 32, 33). Our findings indicate that MUC1 expression in the tumor plays a key role in maintaining the MDSCs in an immature and highly suppressive state and may partially account for the metastatic nature of MUC1^+^ PDA tumors. Indeed, KCM-tumors grow significantly larger than KCKO-tumors in immune competent syngeneic mice (9, 13) (Figure 3A).

Both COX-2 and IL-10 are implicated in MDSC expansion and activation (15). It is well established that there is a positive feedback between PGE_2_ and COX-2 that redirects the differentiation of human dendritic cells toward stable MDSCs (41, 42). In line with that, our data demonstrates that KCM cells express high levels of COX-2 and IL-10 (Figure 5A) and stimulates the generation of highly suppressive and immature MDSCs, thus highlighting the clinical significance of the study. Furthermore, compared to KCKO, KCM-tumors exhibit a fourfold increase in COX-1, sevenfold increase in PGE synthase-2, and threefold increase in PGE synthase-3 levels (Figure 5C). Thus, we postulate that KCM cells may activate the COX-pathway which in turn contributes to the generation of suppressive MDSCs. These increases in the COX-pathway enzymes were correlated with elevated levels of PGE_2_ in the TCCM from KCM-tumor cells and PGEM (PGE_2_ metabolite) in the tumor and serum of KCM-tumor-bearing mice (Figures 5D–F). Thus, when TCCM from celecoxib-treated KCM cells was used, the MDSCs generated expressed low suppressive factors and high maturation markers similar to the phenotype generated with KCKO TCCM (Figures 6C–F). The PGE synthases along with COX-1 and COX-2 are essential for the synthesis of prostaglandins including PGE_2_ from Prostaglandin H_2_ and are being explored as novel therapeutic targets for treatment of various inflammatory diseases and cancer (43, 44). The central role of COX-2-PGE_2_ feedback in the induction and persistence of MDSCs highlights the potential for its manipulation to enhance or suppress immune responses in cancer (41, 42).

Taken together, the data suggests that tumor-associated MUC1 in KCM cells hinders myeloid maturation and drives the generation and maintenance of an immature, suppressive MDSC population partly via activation of the COX-pathway. [*Of note is that the two cell lines KCM and KCKO are derived from the same triple transgenic PDA mouse model that expresses the KRAS^G12D^ mutation driven by the P48 promoter and are therefore genetically similar* (12) except for the presence or absence of Muc1 (9, 13)].

One of the limitations of this study is that all experiments were conducted using KCM and KCKO cell lines. We were unable to conduct these experiments with other cells because there are no other PDA cell lines that are derived in a Muc1-null background. Although there are other low and high MUC1 expressing cells, none that we know lack MUC1 message completely. Since *in vivo* experiments had to be conducted in immune competent mice, human cell lines could not be used for this study. Nevertheless, to confirm that the effect on MDSC may be regulated by the absence or presence of MUC1, we did conduct some of the same experiments using a melanoma cell line (B16) that completely lacks MUC1 message and protein and is syngeneic for C57/BL6 mice thus avoiding strain-related differences. The B16 cells were stably transfected with full-length human MUC1 (designated B16 MUC1) or empty vector (B16 Neo). Figure S2A in Supplementary Material illustrates high MUC1 expression in B16 MUC1 with undetectable levels in B16 Neo cells. MDSCs were isolated from the BM of B16 MUC1 and B16 Neo-tumor-bearing mice and compared to MDSCs isolated from KCM and KCKO-tumor-bearing mice. Results recapitulate the data observed with KCM and KCKO. First, we observed no significant difference in the total numbers of MDSCs between B16 Neo and B16 MUC1 tumor-bearing mice. Second, significantly higher numbers of monocytic MDSCs and lower numbers of granulocytic MDSCs was detected in B16 MUC1 versus B16 Neo-tumor-bearing mice (Figures S2B–D in Supplementary Material). Finally, compared to MDSCs from B16 Neo-tumor-bearing mice, MDSCs from B16 MUC1 mice expressed significantly higher levels of iNOS (Figures S2E–G in Supplementary Material) and Arg-1 (Figures S2H–J in Supplementary Material). These data confirm that the effect on MDSC maturation and function may be regulated by the absence or presence of MUC1.

The effect of COX-2 inhibition on MDSC function is not surprising as this has been established with other tumor types (45). Our observations indicate that KCM cells express higher levels of COX-2 and secrete higher levels of PGE2 than KCKO cells (Figures 5A,D–F). Thus, we hypothesize that the induction of the highly suppressive and immature MDSCs induced by KCM cells may be via the up regulation of the COX-pathways and synthesis of PGE_2_. Our upcoming studies will further our understanding of the underlying mechanisms by which the MUC1 glycoprotein regulates the COX-2 pathway in PDA and examine the effects of shed MUC1 on MDSC maturation and function.

In human PDA, MUC1, and COX-2/PGE_2_ pathways are highly activated and patients have high levels of MDSCs in the tumor, spleen, and blood, thus our data has high clinical relevance as future therapies can be designed to target MUC1 and COX-2 signaling. Targeting MDSCs along with a cancer antigen greatly improves the efficacy of the cancer vaccine (46, 47) and in this regard, we have previously reported that simultaneous targeting of MUC1 and COX-2 in a spontaneous model of PDA was highly effective in stalling the progression of PanIN lesions to adenocarcinomas and in inhibiting invasive disease (48). Data from this study furthers our understanding of the mechanisms by which the combination treatment was effective and provides further rationale for designing clinical trials targeting MUC1 and COX signaling pathways to overcome cancer mediated immune suppression.

Clearly, for the present study to have clinical relevance, these findings have to be validated using patient samples. We have recently published that MUC1 was detected in the circulation of pancreatic cancer patients in a stage-dependent manner (49). Future studies will focus on determining levels of MDSCs and T-regulatory cells in patients with MUC1^+^ and MUC1^−^ PDA.

## Authors Contribution

Amritha Kidiyoor designed all experiments, conducted all experiments, and drafted all versions of the manuscript. Jorge Schettini helped in the design of flow cytometry experiments, helped conduct the first few sets of flow cytometry experiments, helped draft versions of the manuscript and revised manuscript. Dahlia Marie Besmer helped in the design of the *in vivo* study, helped in injecting tumor cells in mice, and harvesting of bone marrow and spleen, helped conduct experiments on MDSCs collected from tumor-bearing mice and edited the manuscript. Stephen Lee Rego helped in the design of the *in vivo* study and protein work (Westerns and ELISAs), helped in harvesting of bone marrow cells from mice, helped conduct experiments on MDSCs collected from tumor-bearing mice, helped conduct Western blot and ELISA experiments and edited the manuscript. Sritama Nath helped conduct experiments on MDSCs collected from tumor-bearing mice, helped conduct a Western blot experiment and edited the manuscript. Jennifer Marie Curry helped in harvesting of organs from tumor-bearing mice, helped in setting up flow cytometry experiments, and edited the manuscript. Lopamudra Das Roy helped in harvesting of organs from tumor-bearing mice and edited manuscript. Didier Dréau helped in the design of ELISA experiments and has provided intellectual input throughout preparation of manuscript along with editing and revising manuscript. Pinku Mukherjee, PI of the lab and project, was involved every step of the way in planning of experiments, analyzing results, and preparation of manuscript.

## Conflict of Interest Statement

The authors declare that the research was conducted in the absence of any commercial or financial relationships that could be construed as a potential conflict of interest.

## Supplementary Material

The Supplementary Material for this article can be found online at http://www.frontiersin.org/Journal/10.3389/fimmu.2014.00067/abstract

## Abbreviations

Arg-1, arginase 1; BM, bone marrow; BMDCs, bone marrow derived cells; COX-2, cyclooxygenase 2; iNOS, inducible nitric oxide synthase; KCKO, PDA cell line without MUC1; KCM, PDA cell line expressing MUC1; MDSCs, myeloid derived suppressor cells; MUC1 (human) Muc1 (murine), Mucin 1; NO, nitric oxide; PDA, pancreatic ductal adenocarcinomas; PGE2, prostaglandin E2; TCCM, tumor cell conditioned media; TDSFs, tumor derived soluble factors.
