# Supplementary material for: Pancreatic Cancer Cells Isolated from Muc1-Null Tumors Favor the Generation of a Mature Less Suppressive MDSC Population
Source: Front Immunol. 2014 Feb 24;5:67. doi: 10.3389/fimmu.2014.00067 (PMC3932420; doi:10.3389/fimmu.2014.00067)
Supplement: Supplementary file 1 [file 76664_Mukherjee_Presentation1.ZIP › Supplementary Material.pdf]

Supplementary Material

Pancreatic cancer cells isolated from Muc1-null tumors favor the generation of a mature less suppressive MDSC population

Supplementary Figure 1 Characterization of maturation and suppressive markers on MDSC subsets: BM cells from in vitro cultures or from tumor bearing mice were collected and stained with anti-Gr1, anti-CD11b, anti-Ly6C, anti-Ly6G, anti-CD11c, anti-CD115, anti-iNOS and anti-Arg-1 antibodies and analyzed by flow cytometry. A) iNOS expression on Gr1+CD11b+ BM cells induced by KCM and KCKO cells; B) Arg-1 expression on Gr1+CD11b+ BM cells induced by KCM and KCKO cells. Histogram from 1 representative experiment out of 4 is shown; C&D) CD11c expression on CD11b+Ly6C+Ly6G- and CD11b+Ly6C+Ly6G+ BM cells from tumor bearing mice; E&F) CD115 expression on CD11b+Ly6C+Ly6G- and CD11b+Ly6C+Ly6G+ BM cells from tumor bearing mice; G&H) Percentage of iNOS+ cells on CD11b+Ly6C+Ly6G- and CD11b+Ly6C+Ly6G+ BM cells from tumor bearing mice; I&J) Percentage of Arg-1+ cells on CD11b+Ly6C+Ly6G- and CD11b+Ly6C+Ly6G+ BM cells from tumor bearing mice. Percentages are not absolute numbers. Mean and standard error are shown. *p<0.05, **p<0.01 and ***p<0.001 for statistically significant differences from control (3T12) levels unless indicated by line above the bars

Supplementary Figure 2 Characterization of MDSCs in B16.Neo and B16.MUC1 tumor bearing mice: C57BL/6 mice (n=4) were injected in the flank with $1x10^6$ 3T12, B16 neo, B16 MUC1, KCM and KCKO cells. Upon sacrifice, bone marrow was harvested. BM cultures were established as previously described. BMDCs were stained with anti-Gr1, anti-CD11b, anti-Ly6C, anti-Ly6G, anti-iNOS and anti-Arg1 antibodies and analyzed as described previously. A) Western blot analysis and quantitative densitometry of MUC1 protein expression in whole cell lysates from B16.Neo and B16.MUC1 cells. MUC1 protein expression was normalized for β-actin protein expression. Western blotting for MUC1 and β-actin protein expression was carried out 3 times. B) Percentage of $Gr1^+CD11b^+$ cells. C&D) Percentage of $CD11b^+Ly6C^+LyG^-$ (C), and $CD11b^+Ly6C^+Ly6G^+$ (D) MDSC subsets. E-G) Percentage of $iNOS^+$ cells on $Gr1^+CD11b^+$ (E), CD11b+Ly6C+Ly6G- (F), and CD11b+Ly6C+Ly6G+ (G) MDSC subsets; H-J) Percentage of $Arg1^+$ cells on $Gr1^+CD11b^+$ (H), CD11b+Ly6C+Ly6G- (I), and CD11b+Ly6C+Ly6G+ (J) MDSC subsets. Mean and standard error are plotted. *p<0.05, **p<0.01 and ***p<0.001 for statistically significant differences from control (3T12) levels unless indicated by line above the bars.
