# Supplementary figures and images for: Pancreatic Cancer Cells Isolated from Muc1-Null Tumors Favor the Generation of a Mature Less Suppressive MDSC Population
Source: Front Immunol. 2014 Feb 24;5:67. doi: 10.3389/fimmu.2014.00067 (PMC3932420; doi:10.3389/fimmu.2014.00067)

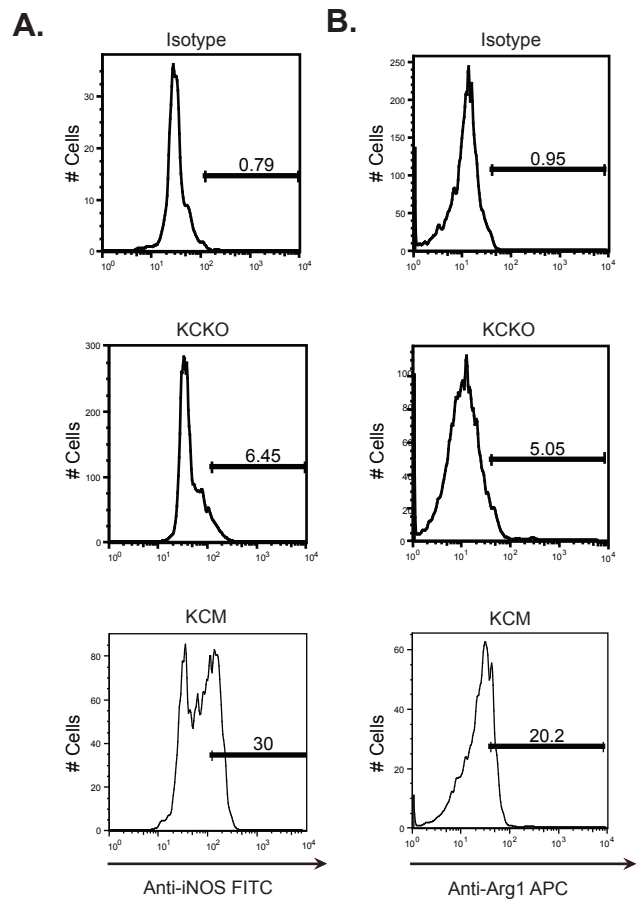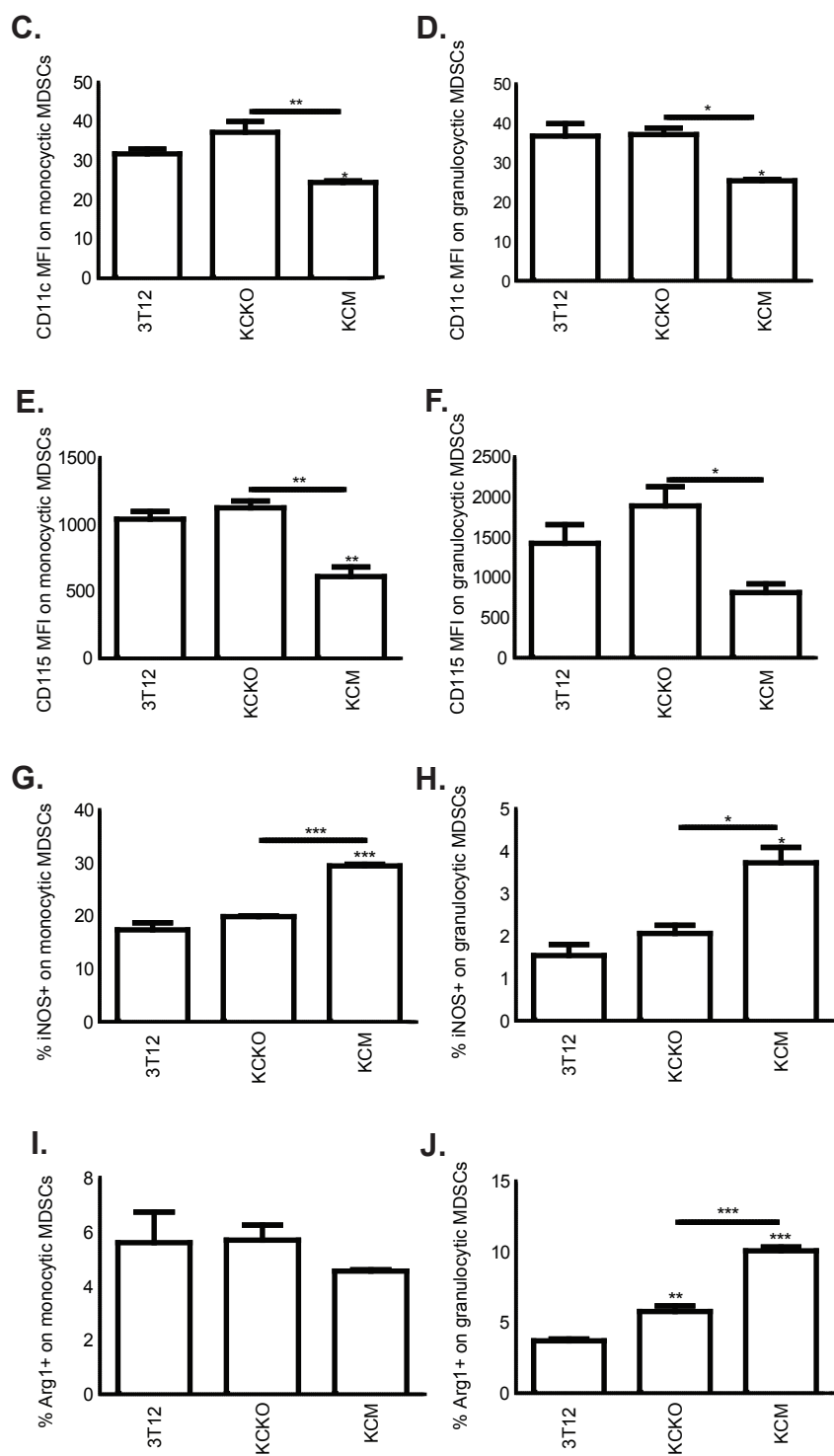

Supplement: Supplementary file 1 [file 76664_Mukherjee_Presentation1.ZIP › Figure S1.pdf]

**A.**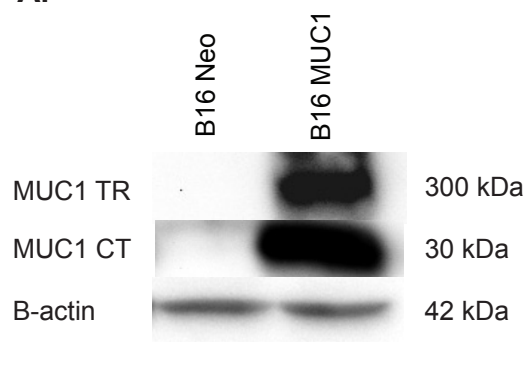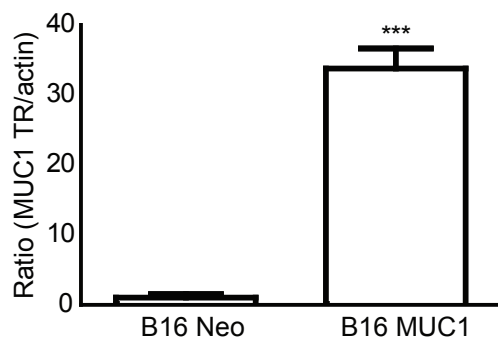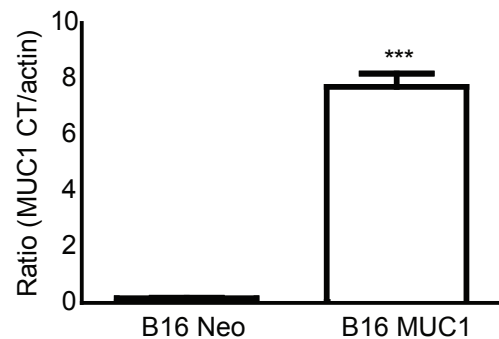**B.**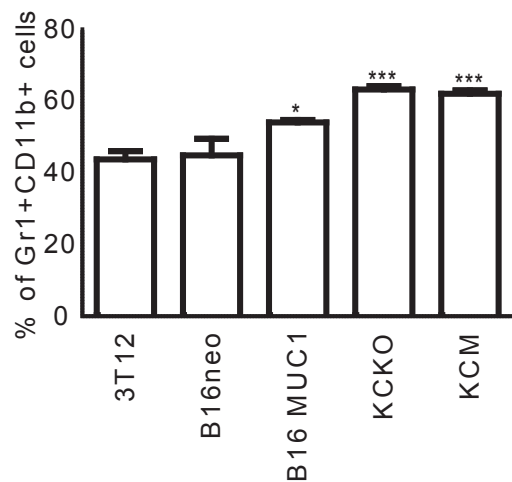**C.**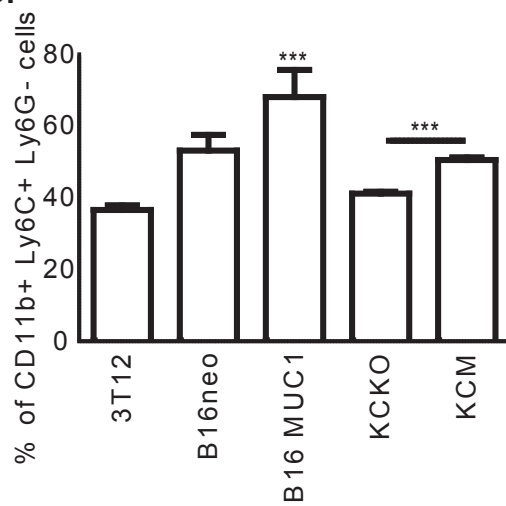**D.**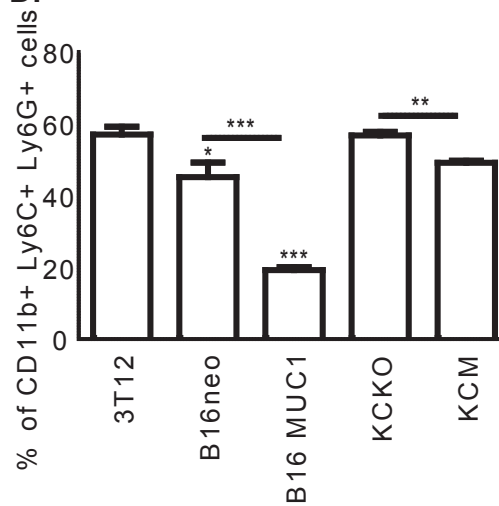**E.**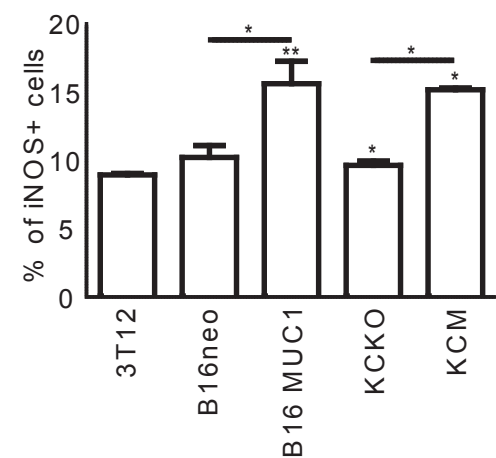**F.**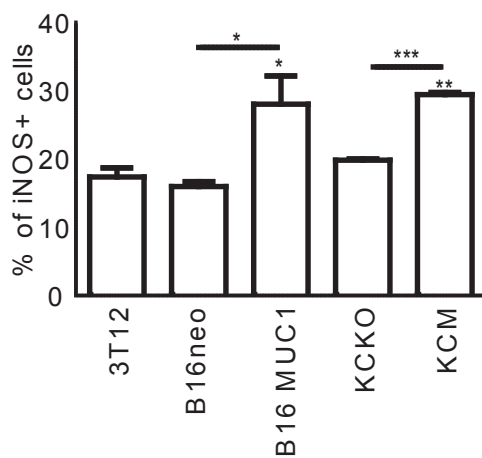**G.**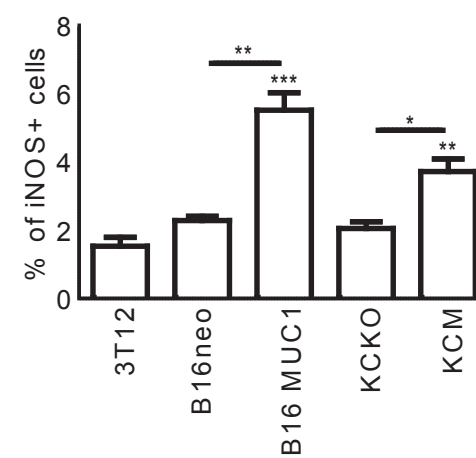**H.**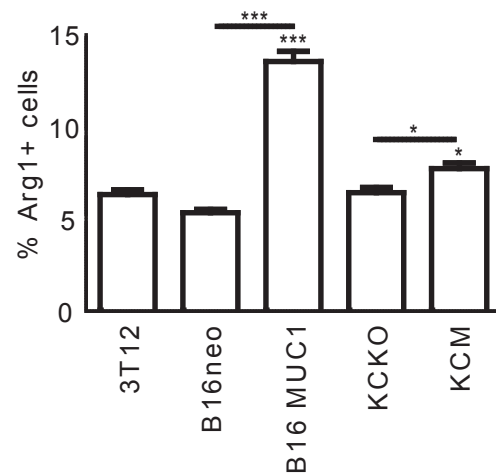**I.**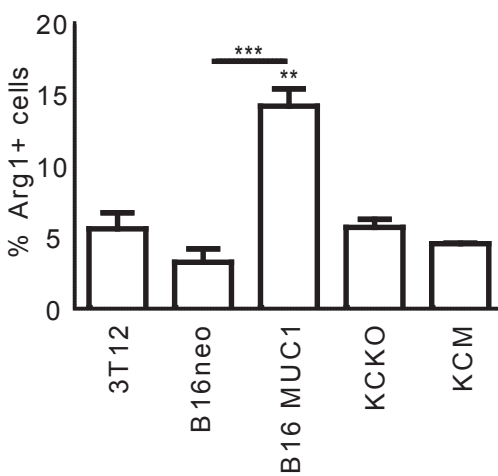**J.**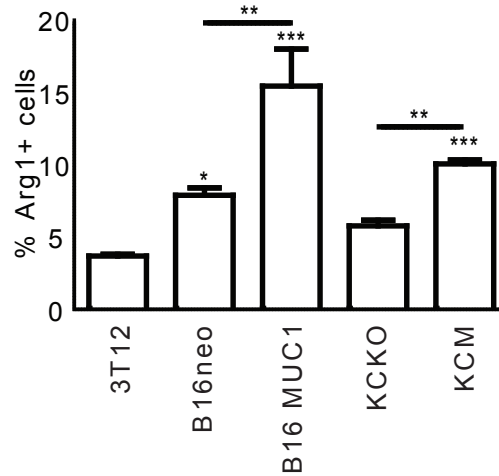

Supplement: Supplementary file 1 [file 76664_Mukherjee_Presentation1.ZIP › Figure S2.pdf]
